# Impacts of recreational cannabis legalization on use and harms: A narrative review of sex/gender differences

**DOI:** 10.3389/fpsyt.2023.1127660

**Published:** 2023-03-10

**Authors:** Justin Matheson, Bernard Le Foll

**Affiliations:** ^1^Translational Addiction Research Laboratory, Centre for Addiction and Mental Health, University of Toronto, Toronto, ON, Canada; ^2^Department of Pharmacology and Toxicology, University of Toronto, Toronto, ON, Canada; ^3^Centre for Addiction and Mental Health, Campbell Family Mental Health Research Institute, Toronto, ON, Canada; ^4^Dalla Lana School of Public Health, University of Toronto, Toronto, ON, Canada; ^5^Department of Psychiatry, University of Toronto, Toronto, ON, Canada; ^6^Institute of Medical Science, University of Toronto, Toronto, ON, Canada; ^7^Acute Care Program, Centre for Addiction and Mental Health, Toronto, ON, Canada; ^8^Department of Family and Community Medicine, University of Toronto, Toronto, ON, Canada; ^9^Waypoint Centre for Mental Health Care, Waypoint Research Institute, Penetanguishene, ON, Canada

**Keywords:** sex, gender, cannabis, legalization, attitudes, prevalence, harms

## Abstract

Legalization of cannabis use for non-medical (recreational) purposes is changing the global cannabis landscape. As attitudes toward cannabis use become more positive and prevalence of use increases in complex ways, concerns emerge about the potential for increased cannabis-attributable harms. Understanding the who, why, and when of this likely increase in cannabis-attributable harms is thus an important public health priority. Both sex and gender contribute to variability in the use, effects, and harms of cannabis and thus sex/gender considerations are important when evaluating the impacts of cannabis legalization. The goal of this narrative review is to broadly discuss sex/gender differences in attitudes toward and prevalence of cannabis use, whether there are sex/gender differences in the impacts of cannabis legalization, and why these sex/gender differences might exist. One of our strongest conclusions is that men have always been more likely to use cannabis than women, yet the sex/gender gap in prevalence of cannabis use has narrowed over time, and this might be partly due to cannabis legalization. The existing evidence suggests that there have also been sex/gender differences in the impacts of legalization on cannabis-attributable harms such as cannabis-involved motor vehicle collisions and hospitalizations, though these results are more variable. The body of literature reviewed has focused almost exclusively on samples of cisgender research participants, and thus future research should encourage inclusion of transgender and gender-diverse participants. More consideration of sex- and gender-based analysis in research evaluating long-term impacts of cannabis legalization is a clear research priority.

## 1. Introduction

Cannabis continues to be one of the most commonly used psychoactive drugs worldwide. The most recent data from the United Nations Office on Drugs and Crime (UNODC) estimate that 209 million people used cannabis in 2020, which represents roughly 4% of the global population (1). The legal status of cannabis has been controversial since the late 1930s, and the past two decades have seen dramatic changes in individual state- and country-level regulation of cannabis worldwide (2, 3). As of November 2022, commercial sale of non-medical (recreational) cannabis is legal at the national level in three countries (Uruguay, Canada, and most recently, Thailand), while an additional four countries have legalized possession and consumption of cannabis for non-medical purposes, with restrictions on sale and distribution (Georgia, Malta, Mexico, and South Africa). In the United States, cannabis is still illegal for any purpose at the federal level, but 21 states, two territories, and the District of Columbia have legalized non-medical cannabis use.

Legalization of non-medical cannabis use has had mixed effects on changes in prevalence of use. For example, one recent systematic review that identified 32 relevant studies found that legalization was associated with an increase in past-month cannabis use among young adults, but may not have had an impact on other cannabis use metrics or in other age groups such as adolescents (4). Data from the most recent National Surveys on Drug use and Health (NSDUH) in the US suggest that daily cannabis use may have increased significantly more than overall use; daily use rose from 0.65 to 2.31% from 2002 to 2020 (a nearly four-fold increase), while past-year prevalence increased from 11.03 to 17.47% over the same time period (5). Similarly, data from the Centre for Addiction and Mental Health (CAMH) Monitor Surveys in the Canadian province of Ontario found that past-year prevalence of cannabis use increased from 11% in 2011 to 26% in 2019, whereas daily use increased from 1 to 6% over the same time period (6). However, data from Monitoring the Future in the US has not seen much of an increase in prevalence of daily cannabis use, which has remained around 5–6% among 12^th^ graders from 2002 to 2022 (7). The evidence is similarly mixed on the extent to which legalization has impacted cannabis-related harms such as prevalence of cannabis use disorder (CUD), cannabis-involved motor vehicle collisions, and cannabis-involved hospitalizations (8–10). Significant heterogeneity in the relationship between cannabis legalization and specific metrics of cannabis use and related harms suggests that legalization has not had a uniform impact across the population in countries that have legal access to non-medical cannabis use.

Sex and gender both have significant impacts on the use and effects of psychoactive drugs, including cannabis. Sex refers to biological attributes and functions of bodies, whereas gender refers to the socially and culturally constructed aspects of self-perception and social organization that shape identity, expression, roles, norms, behaviors, and relations. Both sex and gender are complex, multifaceted constructs, and neither are adequately described by binary frameworks, despite historical emphasis on sex (male, female) and gender (man, woman) as binary traits. While sex and gender are two distinct concepts, in reality, there is an intricate and dynamic relationship between sex and gender and it is challenging to draw a clear line between them; thus, the term “sex/gender” can be used to recognize this entanglement (11). The term “sex/gender” is not meant to conflate the concepts of sex and gender, but rather to acknowledge that we typically lack sufficient information to accurately attribute an observed difference to either sex or gender. For example, if a study finds that prevalence of past-year use of a drug differs between cisgender women and cisgender men (with no potential explanatory variables considered), there is not enough information to label this finding either a “sex difference” or a “gender difference,” as the likelihood of using a psychoactive drug is dependent on both sex-related biological factors and gender-related sociocultural factors. In this case, we would use the term “sex/gender difference.” For the purposes of this review, we will use the term “sex/gender” in cases where we are summarizing data and where there is insufficient evidence to attribute trends in the data to either sex or gender. In order to accurately reflect the evidence that we are reviewing, we will clearly identify whether an individual study analyzed their data with consideration of sex or gender and, where possible, provide details on how sex and/or gender were defined.

Sex/gender differences have been observed in cannabis use prevalence, routes of administration, acute effects, prevalence and severity of cannabis use disorder, and actions of the endocannabinoid system that mediates the effects of cannabis (12, 13). One of the most robust findings is the higher prevalence of cannabis use and cannabis use disorder (CUD) among men, compared to women (to be discussed in more detail in the body of this review). Nearly all of this research has involved cisgender men and women and used a binary man–woman, boy–girl, or male–female comparative framework, though there is a small and growing literature documenting cannabis use attitudes and harms among gender minorities (i.e., transgender and non-binary or gender-diverse individuals). As this manuscript will focus primarily on potential gendered mechanisms driving differences in cannabis-related outcomes, here we present a brief overview of sex differences in responses to cannabis to provide some context to the interested reader. There is a robust literature in animal models demonstrating that female rodents are more sensitive to the effects (e.g., motor, analgesic, and reinforcing effects) of cannabinoid drugs such as Δ-9-tetrahydrocannabinol (THC), the primary intoxication component of cannabis (13). These sex differences in rodents are often attributed to the actions of gonadal hormones such as estradiol; for example, estradiol influences analgesic effects of THC (14) and density of cannabinoid receptors in the mammalian brain (15). Importantly, in rodent models, there is a notable sex difference in metabolism of THC, where females metabolize THC primarily to a psychoactive metabolite, while males metabolize to a variety of mainly non-psychoactive metabolites (16). However, this evidence has not translated so clearly to humans; while multiple studies have found evidence of human sex differences in some cannabis-related outcomes using experimental designs, there is considerable heterogeneity in results, and human sex differences appear to be heavily dependent on factor such as THC dose, route of administration (e.g., smoked vs. oral), and participants’ past experience with cannabis (13, 17).

Given the significant heterogeneity in the impact of cannabis legalization and the robust evidence demonstrating an impact of both sex and gender on cannabis use and related harms, the present narrative review aimed to do four things: (1) describe sex/gender differences in cannabis-related attitudes/perceptions and cannabis legalization support; (2) describe sex/gender differences in cannabis use prevalence and how cannabis legalization impacted cannabis use; (3) present an overview of how cannabis legalization may have impacted cannabis use during pregnancy and parenthood, which are particularly salient gendered life course factors; and (4) present an overview of the scope of evidence that suggests sex/gender may have impacted the effects of cannabis legalization on changes in cannabis-related harms. We took a narrative approach with this review in order to focus more on understanding why these sex/gender differences exist.

## 2. Sex/gender differences in attitudes toward cannabis use and support for cannabis legalization

### 2.1. Sex/gender differences in cannabis use attitudes and risk perceptions

Women tend to have more negative attitudes toward cannabis use than men. For example, in a sample of 1,713 Canadian undergraduate students, women had lower odds (odd ratio [OR] = 0.66) of having favorable attitudes toward cannabis acceptability compared to men (note that sex was used as a proxy variable for gender in this study) (18). Similarly, in a survey of 507 adolescents in Ireland that investigated gender differences, boys were more likely than girls to perceive cannabis as a safe substance (OR = 2.02) and less likely to perceive that cannabis use was a big problem for Irish teenagers (OR = 0.53) (19). In a large national survey of Norwegian university and college students (*n* = 49,688), a gender difference was found among respondents not reporting cannabis use: 40.9% of men perceived cannabis as no/low risk compared to only 16.4% of women (20). An analysis of 2002–2018 data from the National Surveys on Drug Use and Health (NSDUH) in the US (*n* = 949,285) suggested that gender differences in cannabis perceptions may be age-dependent (21). In adolescents aged 12–17 years, there was little evidence of a gender difference in cannabis perceptions, but among adults aged 18+ years, perceiving cannabis as low-risk was more common among men and perceiving cannabis as high-risk was more common among women (21).

One interesting note from the analysis of 2002–2018 NSDUH data in the US is that the gender gap in cannabis risk perceptions did not seem to change over time. While the prevalence of risk perception of cannabis varied by gender when data were aggregated or viewed cross-sectionally, time trend analysis found that the gender gap in risk perception did not change over time, with the exception of a greater decline over time in high-risk perception of cannabis in men in the 50+ age group (21).

### 2.2. Sex/gender differences in support of cannabis legalization and intentions to use cannabis if legalized

Men tend to have greater support for cannabis legalization, whereas women are less likely to support legalization. For example, a survey of 2,190 adults (aged 18+ years) in Michigan found that female gender was associated with lower odds of supporting cannabis legalization (OR = 0.46) (22). The authors of this survey explored reasons for supporting or opposing legalization using qualitative methods and found some gendered differences—women tended to cite potential medical benefits or increasing product safety as reasons for supporting legalization, while men tended to cite personal freedom (23). Other US studies have similarly found an association between male sex or gender and greater support for cannabis legalization (24, 25). The same trend has been observed in Norway (20), Ireland (19), New Zealand (26), and Malaysia (specific to decriminalization of medical cannabis) (27), while another study found that women in the Caribbean were more likely to support full prohibition of cannabis (28).

One study evaluated gender differences in trends in cannabis legalization support over time. The authors found that, while the overall proportion of US adults in favor of cannabis legalization has increased steadily over time (from just over 10% in 1969 to nearly 60% in 2016), gender did not have a significant impact on this trend (29). In this analysis (General Social Survey data spanning the years 1974 to 2016), women were consistently less likely to support legalization than men, though the difference was of small magnitude for most years and did not meaningfully change over the four decades (29). This seems to align with the previously discussed finding that temporal trends in cannabis risk perception over time were not impacted by gender, even though risk perceptions varied by gender when viewed cross-sectionally.

One study was identified that included gender minority respondents. In a survey of young adults who identified as sexual or gender minorities in Chicago (*n* = 1,114), there was a marginally significant effect of gender identity on perception of cannabis legalization, where cisgender men and gender minorities had slightly higher agreement with cannabis legalization than cisgender women (30).

Men are generally more likely to indicate intent to try cannabis if it becomes legal, compared to women. For example, an analysis of sex differences in data from five cohorts (2007 to 2011) of high school seniors in Monitoring the Future (a US national survey) found that, among adolescents not currently using cannabis (*n* = 6,116), female participants were less likely to indicate intention to try cannabis if legalized, compared to male participants (adjusted OR = 0.61) (31). In a study of Norwegian university and college students (*n* = 49,688), among respondents not reporting cannabis use, 13.2% of men intended to try cannabis if legalized, compared to just 5.4% of women (20). Data from multiple cycles of the Australian National Drug Strategy Household Survey (*n* = 23,855 in 2013, 23,749 in 2016, and *n* = 22,015 in 2019) found that male sex was associated with significantly increased odds (adjusted OR = 1.64) of willingness to try cannabis if it were legal (32). One study using data from the 2018 National Cannabis Survey (NCS) in Canada (*n* = 17,089) did not find a significant effect of sex on the odds to try or increase use of cannabis after legalization (after adjusting for province/territory and survey wave), though male sex was significantly associated with increased willingness to try or increase use in the unadjusted model (OR = 1.3) (33). Note that this study used gender as a proxy variable for sex (i.e., the survey variable was gender, but the authors interpreted this as participant sex).

### 2.3. Why are there sex/gender differences in attitudes toward cannabis use and legalization?

An understanding of why sex/gender differences in attitudes toward cannabis use and legalization exist is helpful to contextualize these findings and speculate how things may change over time. A study of 1,820 adolescents (aged 10 to 19 years) in the US suggested that there may be gender differences in determinants of cannabis risk perceptions: peer norms were more strongly related to risk perception in boys, whereas parental norms were more strongly related to risk perception in girls (34). Another study of 1,002 registered US voters (aged 18 to 95 years) suggested that the perceived lack of adequate regulations to prevent cannabis-related harms may play a role; a gender difference in support of cannabis legalization (support initially lower in women) was no longer statistically significant if a reliable roadside test for cannabis-related driving impairment was available (25). In one of the most informative studies on this topic, Elder & Greene (2019) used data from the March 2013 Pew Research Center Political Survey (in the US) to test a series of hypotheses for the gender gap in support of cannabis legalization (35). In contrast to their hypotheses, parenthood was not a significant factor in predicting the gender gap in cannabis legalization support and increased religiosity among women did not fully account for the gender gap, despite being a significant predictor of cannabis legalization attitudes. Instead, the results of this study found that lifetime cannabis use was a highly significant predictor of favorable attitudes toward cannabis legalization, and this did explain the gender gap in attitudes (35). This is in line with other literature demonstrating that prior use of cannabis is one of the most robust predictors of legalization support (22, 31, 36, 37). As we will discuss in the next section, cannabis use is significantly more prevalent among men than women, which is likely at least in part due to stigmatization of women’s cannabis use and sex/gender differences in opportunities to use cannabis.

### 2.4. Interim summary

Taken together, women appear to have more negative attitudes toward cannabis use, greater perception that cannabis use is harmful, and less support for cannabis legalization, and this appears to be true across multiple countries in North America, the Caribbean, Europe, and Asia. The negative association between female gender and support of cannabis use and legalization is likely due to a complex set of sociocultural factors, including adherence to traditional feminine gender roles, sex/gender differences in determinants of risk perceptions, and sex/gender differences in cannabis use. Though the data are still very limited, it appears that there have not been dramatic changes in the sex/gender gap in cannabis risk perception or support of cannabis legalization over time. However, to the best of our knowledge, no study has directly tested for an effect of cannabis legalization on sex/gender parity in cannabis-related attitudes or perceptions. This kind of effect might be expected if the gender differences in attitudes toward cannabis legalization are related to differences in socialization of girls and boys. For example, since girls have historically been socialized to worry more about moralistic social issues, one could predict that adult women who were socialized during periods of heightened concern toward illicit drug use might have much more negative attitudes toward cannabis use and legalization than younger women who were socialized during periods of increased societal acceptance of cannabis use, whereas this effect might not be observed in men (35).

## 3. Sex/gender differences in cannabis use prevalence and the impact of cannabis legalization on cannabis use

### 3.1. The sex/gender gap in cannabis use prevalence: Historical trends

Cannabis use has historically been significantly more prevalent among men than women, yet the sex/gender gap in prevalence has changed over time. Early data from the US National Alcohol Surveys suggested a gender convergence of past-year cannabis use from 1984 to 2000, which was largely driven by a greater decline in use among men overall, and a notably steep increase in past-year cannabis use in women aged 18 to 25 years between 1995 and 2000 (38). Data from the National Youth Risk Behavior Survey (school-based surveys of high school students in the US) from 1999 to 2013 similarly found evidence of sex converge in prevalence of past 30-day cannabis use over that time period (39). In contrast, one study using data from the National Survey on Drug Use and Health (2002–2014) actually found a widening of the gender gap in prevalence of past-year cannabis use, which was primarily due to an increase in use among men of lower income from 2007 to 2014 (40).

Chapman et al. conducted a systematic review and meta-analysis of studies examining birth cohort changes in the sex/gender gap in cannabis use prevalence in North America, Europe, and Oceania (41). Note that the authors did not specify whether the reviewed studies analyzed data with respect to sex or gender, and the authors use the terms sex and gender interchangeably in this article, so we retain the term “sex/gender” when discussing these findings here. Of the 22 studies included in the systematic review, 10 found evidence of sex/gender convergence in at least one indicator of cannabis use (e.g., past-month, past-year, or lifetime cannabis use), with the majority (7/10) finding that the convergence was due to a greater increase in use among women compared to men (41). Eleven studies found no evidence of changes in sex/gender differences in cannabis use over time, while a single study found that there was evidence of sex/gender divergence in prevalence of use driven by increased use among men (41). In the quantitative synthesis and meta-analysis, the pooled cannabis use sex/gender ratio varied from a high of 2.0 (men to women) in the 1941–1945 birth cohort to a low of 1.3 in the most recent birth cohort (1991–1995), and the meta-regression indicated that the decline in the cannabis use sex/gender ratio over time was linear and statistically significant (41).

The most recent data available, as reported in the 2022 UNODC World Drug Report, found that the gender gap in past-month prevalence of cannabis use in the US declined from 2002 to 2020, from a high of approximately 2.125 men to women reporting past-month use in 2007 to a low of approximately 1.25 men to women reporting past-month use in 2020 (1).

### 3.2. Sex/gender differences in cannabis use prevalence: Recent data

Cannabis use prevalence data from the Canadian Cannabis Survey (CCS; yearly data available from 2017 to 2022, available disaggregated by sex) and the US NSDUH (yearly data from 2017 to 2020, available disaggregated by gender) are presented in **Table** **1** (past-year prevalence) and **Table** **2** (prevalence of daily use). The CCS is an annual online cross-sectional survey of Canadians aged 16 years or older that has been administered since 2017, with the goal of evaluating the impact of the *Cannabis Act* on cannabis-related metrics in Canada (42–47). The NSDUH is a much longer-standing annual cross-sectional survey in the US, which has been running since 1971 and includes Americans aged 12 years or older (5). Overall, the past-year data (**Table** **1**) show similar prevalence in Canada and the US that seems to be rising over the past 4–5 years, with slightly higher prevalence each year in Canada and prevalence consistently higher in male respondents compared to female respondents. Daily use data are not comparable between Canada and the US because the weekly frequency data are restricted to respondents reporting past-year use in the CCS, whereas the NSDUH asks weekly frequency of all respondents. Nevertheless, trends in the data support increasing daily use of cannabis in both male and female respondents over time in the US, with less consistent trends in Canada.

**Table 1:** Past-year prevalence of cannabis use from the Canadian Cannabis Survey [data available from (42–47)] and the US National Surveys on Drug Use and Health [data available from (5)].

	2017	2018	2019	2020	2021	2022
CCS						
Males	26.1 [24.7–27.5]	26.5 [25.3–27.7]	28.6 [27.4–29.9]	30.7 [29.3–32.1]	28.6 [27.3–30.0]	29.7 [28.3–31.2]
Females	17.5 [16.3–18.8]	17.6 [16.7–18.7]	20.7 [19.6–21.8]	23.4 [22.2–24.6]	22.1 [20.9–23.3]	24.7 [23.5–26.1]
NSDUH						
Men	17.66 [16.90–18.44]	18.50 [17.80–19.23]	20.48 [19.81–21.17]	19.51 [18.26–20.84]	-	-
Women	12.49 [11.99–13.01]	13.56 [12.93–14.22%]	14.86 [14.25–15.49]	15.54 [14.47–16.67]	-	-

Data are presented as percentages (percent of total respondents endorsing past-year use) [95% CI]. Note that the terms “males” and “females” are used here to be consistent with CCS language.

**Table 2:** Prevalence of daily cannabis use from the Canadian Cannabis Survey [data available from (42–47)] and the US National Surveys on Drug Use and Health [data available from (5)].

	2017	2018	2019	2020	2021	2022
CCS						
Males	19.4 [17.2–21.9]	20.2 [18.2–22.4]	18.6 [16.8–20.6]	21.0 [18.9–23.2]	21.1 [18.9–23.4]	20.8 [18.6–23.2]
Females	16.8 [14.0–20.1]	16.3 [14.1–18.7]	16.1 [14.1–18.3]	13.7 [11.8–15.8]	16.2 [14.2–18.5]	15.6 [13.6–17.8]
NSDUH						
Men	2.00 [1.76–2.26]	2.26 [2.06–2.48]	2.52 [2.27–2.80]	2.78 [2.39–3.24]	-	-
Women	1.11 [0.96–1.28]	1.23 [1.07–1.40]	1.50 [1.35–1.67]	1.86 [1.60–2.16]	-	-

Data are presented as percentages [95% CI]. Note that CCS data represent percentage of daily use among respondents reporting past-year use of cannabis, whereas NSDUH data represent percentage daily cannabis use among all respondents.

While research on cannabis use among gender minorities is more limited, some evidence suggests higher use among transgender men compared to transgender women, paralleling the findings observed in cisgender adults. For example, in a US study of transgender adults (*n* = 1,210), 31.3% of transgender men reported past 3-month cannabis use compared to 19.0% of transgender women (48).

### 3.3. Sex/gender differences in the impact of cannabis legalization on cannabis use

A recent systematic review of post-legalization changes in adolescent and young adult cannabis use found mixed evidence for an impact of sex/gender, but overall, the results suggested that increase in post-legalization consumption was higher in girls and young women (4). This review identified eight studies that examined sex/gender influences on change in cannabis use; five found evidence that the increase was greater in girls/women. One large study of US undergraduate students in states that did enact non-medical cannabis legalization (*n* = 234,669 in seven states) or did not (*n* = 599,605 in 41 states) found that past 30-day cannabis use increased more among students in states with legal non-medical cannabis, and when data were disaggregated by gender, this effect was larger among young women (OR = 1.29 for women, 1.12 for men) (49). A longitudinal study of 563 young adults (aged 18 to 24 years) followed from 2015–2016 to 2019 found complex sex by time and sex by legalization interactions for changes in cannabis use over the study period—male participants’ cannabis use decreased over time, but with a slight (non-significant) increase in use following legalization, whereas female participants’ cannabis use increased over time, with a slight (non-significant) decrease in use following legalization (50). An analysis of sex differences in data from a repeated cross-sectional survey of Colorado high school students (*n* = 26,019 in 2013 to *n* = 15,970 in 2015) found a slightly (non-significant) decrease in male participants’ past 30-day cannabis use, whereas there was a (marginally significant) increase in female participants’ cannabis use (51). A similar analysis of sex differences in data from a repeated cross-sectional survey of undergraduate students at Washington State University (total *n* = 13,335 spanning 2005 to 2015) found that female participants had a greater increase in cannabis use following non-medical cannabis legalization than male participants (52). Another similar study of sex differences using repeated cross-sectional survey data (the California Healthy Kids Survey from 2010–2011 to 2018–2019, total *n* = 3,330,912) found that non-medical cannabis legalization was associated with a greater increase in both lifetime (OR = 1.17) and past 30-day (OR = 1.18) cannabis use in female participants compared to male participants (53). In contrast, one study of gender differences using data from the National Cannabis Survey in Canada found a statistically significant increase in cannabis use in men, but not women, in the year following legalization (2018 to 2019) (54). Finally, two studies using US data did not find a significant impact of sex/gender on changes in cannabis use related to legalization (55, 56).

One study was identified that explored the relationship between cannabis legalization and odds of using cannabis among sexual and gender minority youth. Data from the 2017 LGBTQ National Teen Survey (*n* = 10,027 youth in the US) found that residing in a state where cannabis is legal for non-medical purposes was associated with significantly increased odds (OR = 1.50) of current cannabis use, compared to residing in states with no legal access (57). Furthermore, experiences of sexual or gender minority victimization were associated with greater odds of both lifetime (OR = 1.98) and current (OR = 1.99) cannabis use.

### 3.4. Why are there sex/gender differences in cannabis use prevalence?

There are numerous potential explanations for the observation of greater cannabis use among men, mostly rooted in gender norms, roles, and relations (58). One of the most likely explanations for sex/gender differences in cannabis use relates to early opportunities to use cannabis—in general, boys tend to have more opportunities to use cannabis than girls (59–62). Boys may be less supervised by parents, more likely to engage in outdoor activities that put them at increased exposure to drug use opportunities, or more likely to affiliate with older peers who have access to cannabis (59). It should be noted that this finding is not necessarily uniformly true across cultures (61), and since gender as a construct changes over time, changes in gender roles, norms, and relations may reduce this apparent gender difference in opportunities to use cannabis. Quantitative studies have found that cannabis use is positively associated with adherence to traditional masculine gender norms (63, 64), whereas adherence to traditional feminine gender norms tends to have a negative association with cannabis use (65, 66), and these relationships seem to be true for both boys and girls. For both boys and girls expressing masculinity, using cannabis might be a way to demonstrate masculine “toughness” (67, 68). In support of this idea, a handful of qualitative studies have found that adolescents and adults may “do gender” by engaging in cannabis use, i.e., using cannabis to reinforce masculinity or to resist femininity (69–72). On the other hand, qualitative work has demonstrated how gender norms might shape cannabis use, e.g., in a study of Canadian youth, regular use of cannabis by girls was perceived as inappropriate, whereas use by boys was perceived as cool (70). Taken together, these findings suggest that sex/gender differences in cannabis use prevalence are likely driven by a complex interplay of gender influences on initial opportunities to use cannabis (typically higher in boys), the use of cannabis to assert or reinforce masculinity (which would especially increase use among men and boys), and the stigma and negative attitudes toward women and girls using cannabis (which would reduce the likelihood of use among women/girls).

### 3.5. Interim summary

Cannabis use has historically been and continues to be more common among men than women, yet the sex/gender gap in use prevalence has clearly narrowed over time. Based on the available evidence, it seems that legalization of non-medical cannabis has contributed to a narrowing of the sex/gender gap, where the association between legalization and increased cannabis use is observed more consistently among women and girls. However, it is clear that sex/gender influences on cannabis use prevalence are complex and legalization is likely just one of many factors that influences the relationship. Changes in gender norms, roles, and relations over time will likely continue to shift the sex/gender gap in prevalence of cannabis use, independently of legalization.

## 4. The impact of cannabis legalization on cannabis use during pregnancy and parenthood

### 4.1. Cannabis use during pregnancy

Use of cannabis during pregnancy is a particularly salient gendered issue that is associated with significant medical and sociocultural stigma. Pregnant people who use cannabis may continue to do so during pregnancy for perceived health benefits (e.g., anti-nausea effects, pain and stress relief, sleep) and lack of clear information about the harms of gestational cannabis exposure (73, 74). As noted earlier, women’s drug use is already stigmatized, and the increased stigma faced by pregnant people who use cannabis is a likely barrier to seeking information about prenatal cannabis use from healthcare providers, especially since cannabis use during pregnancy may be associated with legal repercussions such as interactions with child protective services (73, 75).

To date, evidence is mixed with regard to effects of gestational exposure to cannabis. While there is currently no direct evidence of cannabis as a teratogen (a compound that causes disturbance of fetal development) (76), observational human studies have raised concerns about long-term effects of cannabis exposure *in utero* (77). Lower mean birth weight in infants seems to be the most consistent effect of gestational cannabis exposure (76, 78, 79), as demonstrated in a recent meta-analysis (OR = 1.77) which also found a mean difference of 109 grams less in infants exposed gestationally to cannabis (76). Other studies have found potential neurobehavioral consequences of gestational exposure, including effects on cognition and aggression, though these studies have been criticized for not adequately controlling for relevant confounds such as co-use of other psychoactive drugs (e.g., alcohol, nicotine, opioids), maternal mental health disorders, and environmental factors such as poverty (75, 79, 80).

Wilson & Rhee recently systematically reviewed literature that evaluated the impact of cannabis legalization in the US on cannabis use during pregnancy and perinatal outcomes (81). Based on 16 identified studies, the authors concluded there was sufficient evidence to suggest that cannabis legalization caused an increase in cannabis use, CUD, and CUD treatment admissions during the preconception, pregnancy, and postpartum periods (81). Furthermore, based on six studies, there was some mixed evidence regarding cannabis legalization effects on perinatal and postnatal outcomes such as low birth weight and preterm birth (81). Data from one study in British Columbia suggest similar results in Canada. Legalization of cannabis in Canada was associated with significantly greater odds (adjusted OR = 1.71) of cannabis use during the preconception period, though the OR associated with the pregnancy period was not significant (82).

### 4.2. Cannabis use during parenthood

Cannabis use during parenthood is another salient gendered issue, where cisgender women/mothers experience greater stigma than cisgender men/fathers. Despite the stigma toward cannabis use by parents, some evidence suggests that cannabis may actually help to improve parent–child relationships by reducing parental stress, substituting for other drugs, or positively influencing parenting style (75).

The systematic review by Wilson & Rhee found some evidence of an increase in parental cannabis use associated with cannabis legalization, based on five studies (81). This may be at least partly due to increases in parental approval of cannabis use and decreases in perceived harms of adult cannabis use among parents (83, 84). Despite the increased use of cannabis by parents and decreased perceptions of harm of adult cannabis use, findings suggested that parents generally remained concerned about and disapproving of adolescent use of cannabis, regardless of legalization (81). Based on two studies reviewed, Wilson & Rhee (2022) concluded that there was insufficient evidence to determine whether cannabis legalization has had any impact on child abuse or neglect (81).

### 4.3. Interim summary

Taken together, the evidence suggest that cannabis legalization has led to increases in cannabis use before, during, and after pregnancy and during parenthood, which is likely related to general increases in adult cannabis use associated with legalization and increased approval of adult cannabis use among parents. However, so far, the evidence suggests a mixed relationship between cannabis legalization and adverse pregnancy outcomes, and no evidence that legalization has led to an increase in child abuse or neglect. Further longitudinal work is needed to disentangle the relationships between cannabis legalization, increased use of cannabis among pregnant people and parents, and potential adverse postnatal outcomes. At the same time, counseling of pregnant people and parents who use cannabis should shift to a harm reduction approach to avoid reinforcing barriers to disclosing personal cannabis use and encourage evidence-based discussions of potential harms and benefits of cannabis use during pregnancy and parenthood (75).

## 5. Sex/gender differences in the impact of cannabis legalization on cannabis-related harms

### 5.1. Cannabis use disorder

Men are more likely than women to meet criteria for CUD and typically have greater severity of CUD symptoms (85–88). For example, in the 2020 NSDUH, 6.03% of male respondents were estimated to have a CUD, compared to 4.08% of female respondents (5). Yet, women may escalate their use of cannabis faster than men (e.g., fewer years between age of first use and age of first CUD symptom) (85, 87, 89, 90), an observation that has been termed the “telescoping phenomenon.” It should be noted that telescoping research has been criticized for the significant male bias in foundational research (which was overwhelmingly based on data from cisgender men) and interpretation of results (which employed a framework that implicitly positioned men’s substance use as normative and women’s substance use as deviant or more pathological) (91). Nevertheless, a significant body of literature has documented sex/gender differences in CUD presentation, such as more frequent or severe mood symptoms (including suicidality and general psychological distress) in women with CUD (92–95) and greater “hazardous” or higher-risk cannabis use (e.g., larger quantities or longer episodes of use, use prior to engaging in activities like driving) in men with CUD (85, 96, 97).

In addition to the previously discussed gendered factors that influence attitudes toward and prevalence of cannabis use, there are likely significant biological factors that influence CUD prevalence and severity. For example, a significant body of literature in animal models has found that female rodents are more sensitive to the reinforcing and rewarding effects of cannabinoids like THC than male rodents (13). Sex differences work from our group at the Centre for Addiction and Mental Health (CAMH) has found that, compared to male participants, female participants tend to smoke less cannabis under placebo-controlled laboratory conditions and have lower concentrations of THC in blood, yet experience similar subjective cannabis high, suggesting that women may need lower doses of cannabis to experience the same high as men (98, 99). Other human laboratory studies have found that, at the same dose of THC, female participants may experience greater subjective effects of cannabis than male participants, including some positive subjective effects associated with addiction liability (100, 101). Interestingly, some studies in rodent models have found that females develop tolerance to certain effects of THC faster than males (102, 103). Taken together, the available animal and human evidence suggests that female sex may be associated with reinforcing and/or rewarding effects of cannabis at lower doses than male sex, which in combination with faster tolerance, could lead to faster escalation of use in addiction-vulnerable individuals assigned female at birth. While these findings are intriguing and may, in part, explain why women tend to escalate use of cannabis faster than men, it is important to recognize that sex-related biological influences on responses to cannabis are still not fully understood and that sociocultural factors still play a significant role in determining trajectories of cannabis use.

Limited evidence of cannabis legalization effects on CUD prevalence or severity exists, let alone evidence that considers sex/gender. For example, a recent systematic review including articles up to March 2022 (with a focus on youth and young adults) identified only one relevant study that examined the impact of cannabis legalization on CUD (104). An analysis of US data from the National Survey on Drug Use and Health (2008 to 2016) found an increase in past-year CUD associated with legalization in adolescents aged 12–17 years and adults aged 26+ years, but not young adults aged 18–25 years (105). Data in Canada found a statistically significant increase in prevalence of CUD in young adults aged 18 to 24 years (106). Neither study considered sex/gender differences.

### 5.2. Cannabis-related motor vehicle collisions and other injury

Driving under the influence of cannabis (DUIC) is a behavior that is more common in men than women (107–111). For example, among respondents who reported past-year cannabis use in the 2021 CCS, 26.2% of male respondents reported driving within 2 hours of smoking or vaping cannabis, compared to 13.8% of female respondents (45). This is likely due to a number of factors, including the increased prevalence of cannabis use among men (as previously described), reduced perception of risk of DUIC among men (112, 113), or other characteristics that tend to more common among men than women, such as increased risk-taking (114, 115).

The results are very limited with regard to sex/gender differences in changes in DUIC and collision risk associated with cannabis legalization. One study in the Canadian province of British Columbia used data from drivers treated after motor vehicle collisions at four trauma centers (spanning January 2013 through March 2020) and included sex in their analysis. The authors found that the increased prevalence of moderately injured drivers with a level of THC in blood of at least 2 ng/ml associated with legalization was greatest among male participants (adjusted prevalence ratio = 2.44) (116). An analysis of gender differences in data from the National Cannabis Survey in Canada found that men were more likely than women to report driving within 2 h of using cannabis in the past 3 months both before and after legalization, though the gender difference did not change appreciably from pre- to post-legalization (117). In contrast, a recent systematic review (64 observational studies included) found that legalization of medical cannabis was associated with greater decline in motor vehicle fatalities among male participant than female participants, though they did not identify any studies that examined sex differences in the impact of non-medical cannabis legalization on DUIC or collision risk (118). The lack of consideration of sex in studies of cannabis legalization and DUIC is a significant limitation of this literature.

One study was identified that evaluated the impact of state cannabis laws on self-harm and assault, using US data on commercial and Medicare Advantage health plan beneficiaries from January 1, 2003, to December 31, 2017, and considered sex (119). While no overall effects of cannabis legalization on rates of self-harm or assault were found, there was a significant effect of legalization on self-harm in male participants under 40 years old (119).

Taken together, the limited available evidence suggests that legalization of non-medical cannabis may drive further increases in prevalence of DUIC and collision risk among men. A recent study in the US found that perceived safety of DUIC, but not perceived legality, was significantly associated with DUIC, and that perceived safety mediated the relationship between cannabis legalization and DUIC (120). This speaks to the need for cannabis and driving educational campaigns targeted specifically to young men, who are by the far the most likely demographic to engage in DUIC and be involved in motor vehicle collisions.

### 5.3. Cannabis-attributable hospitalizations

Acute, transient effects of cannabis such as cognitive impairment, psychotomimetic (i.e., psychosis-like) effects, and psychological distress can lead to emergency department (ED) visits, especially in situations of accidental exposure to cannabis or unexpected highs from high-dose cannabis products (3, 8). Data in the US (especially from Colorado and Washington states, which have the longest history of legal access to non-medical cannabis) have identified associations between cannabis legalization and ED visits related to CUD, motor vehicle accidents and other accidental injury associated with cannabis use, head injuries, cyclic vomiting (likely representing an increased incidence of cannabis hyperemesis syndrome), childhood poisonings and accidental pediatric exposures, psychological distress in adults, and burns related to unsafe handling of butane during attempts to isolate THC from cannabis oil (10). Pre-legalization research has general found that more men than women present with cannabis-related ED visits and hospitalizations (94, 121).

Studies assessing the impact of cannabis legalization on cannabis-related ED visits, hospitalizations, and reports to poison control centers have had mixed findings with regard to sex/gender differences. For example, a study using ICD-10 codes from academic medical centers in Boston, Massachusetts (data from January 2012 to December 2019) found evidence of a gender difference; i.e., that legalization was associated with an increase in the ratio of women to men testing positive for cannabinoids upon ED presentation (122). Another study in the province of Ontario that considered gender found that cannabis legalization was associated with significant increases in cannabis-related ED visits, especially among women (123). Some further interesting trends emerged in this study. For example, the initial legalization of cannabis use in October 2018 was associated with an increase in cannabis-related ED visits especially among women aged 45–64 years, whereas the legalization of cannabis edibles in 2020 was most strongly associated with increased cannabis-related ED visits among women aged 18–44 years (123). The authors proposed that the initial legalization effect was due to older adults (especially women) trying cannabis for the first time, whereas the effect of cannabis edible legalization may be related to the increased preference for edible products among younger adults (123). A repeated cross-sectional study in Ontario, Canada, found that legalization of cannabis edibles and commercialization of new cannabis products in February 2020 was associated with a significant increase in ED presentations of cannabis hyperemesis syndrome, which seemed to be a sex-related effect driven by a statistically significant increase in female participants but not male participants (124). In contrast to the previous studies, data from Colorado found that cyclic vomiting presentations to the ED increased in parallel to increases in cannabis use associated with non-medical legalization, and that men who presented to the ED with cyclic vomiting had significantly greater odds (OR = 2.4) of cannabis-related codes than women (note that this study used sex and gender interchangeably) (125). An analysis of sex differences in data from the Canadian province of Quebec found a significant increase in the percentage of substance-related hospitalizations involving cannabis from pre- to post-legalization in male participants aged 10 to 14 years, but not female participants of any age or older male participants (126). Similarly, an analysis of data from the US National Poison Data System that considered sex found that the commercialization of non-medical cannabis was associated with increases in cannabis exposures reported, and the increase was greater among male participants than female participants (127).

Taken together, there appears to be mixed evidence for sex/gender differences in cannabis legalization impacts on cannabis-attributable hospitalizations. A very tentative conclusion is that legalization may have increased cannabis-attributable hospitalizations to a greater extent in women, especially older women, which would be in line with prevalence data showing greater increases in cannabis use among women in the past several years. However, a few studies have found that legalization led to even greater increases among men and boys. Given the Ontario findings of gender by age interactions in the impact of legalization (123), it will be imperative for future studies to monitor trends using an intersectional approach.

## 6. Conclusion

Legalization of non-medical cannabis use has clearly led to changes in the global cannabis landscape, including changes in attitudes toward use, prevalence and patterns of use, the demographics of individuals using cannabis, and in cannabis-attributable harms. There are broad and robust sex/gender differences in the use, effects, and harms of cannabis, and there are likely sex/gender differences in the impacts of cannabis legalization. Women tend to have more negative attitudes toward cannabis use and legalization than men, and this is likely due to a complex interplay of gender roles and norms. While overall attitudes toward cannabis use have become more positive over time and support for cannabis legalization has increased, the sex/gender gap in legalization support may not have changed to a significant extent, suggesting that the underlying gender constructs that influence legalization support have not changed meaningfully. However, there have been significant changes in the sex/gender gap in cannabis use prevalence over time: while men have historically been much more likely than women to use cannabis, this gap has narrowed. The narrowing of the sex/gender gap in cannabis use prevalence is presumably due to changing gender norms and roles, though there is significant evidence that cannabis legalization has played a role in narrowing the gap. There seems to be a significant effect of cannabis legalization on increased use of cannabis before, during, and after pregnancy, and during parenthood, though this (so far) does not seem to be associated with a corresponding increase in adverse pregnancy or early childhood outcomes. Legalization may have further increased the likelihood of cannabis-related motor vehicle collisions, and there seem to be complex sex/gender influences on the impact of legalization on cannabis-attributable hospitalizations.

One important take-away of this review is the need for more robust data on sex/gender differences in cannabis legalizations impacts. Despite a broad body of literature evaluating impacts of legalization, few studies have considered sex or gender. Further, the existing research has focused almost exclusively on cisgender adults, and more work is needed to understand how legalization may have impacted transgender and gender-diverse youth and adults. More consideration of sex and gender in cannabis legalization research will be imperative to fully understand the scope of legalizations impacts worldwide.

## Author contributions

JM conceptualized the manuscript and wrote the first draft. BLF provided guidance and contributed to reviewing and editing the manuscript. All authors contributed to the article and approved the submitted version.

## Funding

JM received financial support from the Centre for Addiction and Mental Health (CAMH) *womenmind*^TM^ community during preparation of this manuscript.

## Conflict of interest

BLF has obtained funding from Pfizer (GRAND Awards, including salary support) for investigator-initiated projects. BLF has some in-kind donation of cannabis product from Aurora and medication donation from Pfizer and Bioprojet and was provided a coil for TMS study from Brainsway. BLF has obtained industry funding from Canopy (through research grants handled by CAMH or University of Toronto), Bioprojet, ACS and Alkermes. BLF has received in kind donations of nabiximols from GW Pharma for past studies funded by CIHR and NIH. He has been a consultant for Shionogi. He is supported by CAMH and a clinician-scientist award from the department of Family and Community Medicine of the University of Toronto and an Addiction Psychiatry Chair from the department of Psychiatry of the University of Toronto. BLF also participated in an advisory board meeting for Indivior and got a grant from Indivior for a clinical trial.

The remaining author declares that the research was conducted in the absence of any commercial or financial relationships that could be construed as a potential conflict of interest.

## Publisher’s note

All claims expressed in this article are solely those of the authors and do not necessarily represent those of their affiliated organizations, or those of the publisher, the editors and the reviewers. Any product that may be evaluated in this article, or claim that may be made by its manufacturer, is not guaranteed or endorsed by the publisher.
